# Essay on the Effects Produced in the Human Body by Unreduced Congenital and Accidental Dislocations of the Femur

**Published:** 1842-04

**Authors:** 


### Art. IX.

*Essai sur les Effets produits dans le Corps Humain par la Luxation congénitale et accidentelle non reduite du Femur.* Par G. Vrolik, m.d. &c.—*Amsterdam*, 1839. 4to, pp. 30, pl. 4.

*Essay on the Effects produced in the Human Body by unreduced Congenital and Accidental Dislocations of the Femur.* By G. Vrolik, m.d. &c.—*Amsterdam*, 1839. 4to, pp. 30, pl. 4.

The records of surgery contain very few observations respecting the changes consequent on dislocations of the femur, nor are these few described with sufficient accuracy and minuteness to render them really valuable. This brief memoir, from the pen of the most renowned pathological anatomist in Holland, furnishes more and better information on the subject than any other work that we have been able to consult; and we regret to be obliged to pass by the very accurate descriptions of the cases of dislocation which he has dissected, and to limit our extracts to the immediate subject of the memoir, namely, the ulterior changes which follow these displacements.

In consequence of an unreduced dislocation of the thigh on to the ilium, he says (p. 10), the vertical position of the body is necessarily disturbed, because in this posture, and in walking, the centre of gravity of the deformed side is no longer coincident with the centre of motion, that is, with the position of the head of the femur in its articulation. The patient, finding this difficulty, endeavours to remedy it by a complete displacement of the relative position of the parts. When at rest he throws the weight of the body on the healthy limb, and thus the hip of that side becomes habitually lower than that of the diseased side, contrary to what one might expect. The shortening of the long-dislocated limb, therefore, is partly real and partly apparent; the former in consequence of the actual displacement of the femur, the latter in consequence of the elevation of that side of the pelvis.

Again, not only are the raised heel and the in-turned toe of the dislocated limb peculiar, but the whole form of the foot is altered; the instep is higher, the hollow of the sole is greater, the os calcis is longer, and its posterior tuberosity has a remarkable projection downwards. The elevation of the heel therefore is by no means an exact index of the amount of shortening of the limb, but is the result of a combination of those distinct circumstances, namely, the dislocation, the elevation of that side of the pelvis, and the prolongation downwards of the os calcis.

As soon as one side of the pelvis drops, the direction of the vertebral column alters, the lumbar vertebræ turn from that side, the dorsal become curved in the opposite direction, and (though less evidently) there is a slight repetition of the same phenomena in the cervical portion of the spine. At the same time the last lumbar vertebra presses with more than usual force upon the top of the sacrum, and produces an unnatural projection of the promontory, and a greater than ordinary inclination downwards of the pelvic axis. The sacrum loses its vertical position between the ilia; the symphysis pubis is no longer exactly opposite the middle of the sacrum; the antero-posterior diameter of the pelvis assumes an oblique direction from side to side, and the whole pelvis becomes oblique* and unsymmetrical, the diseased side being larger than the healthy.

Nor is this all; the shoulders are thrown down to restore the balance of the body lost by the obliquity of the pelvis; the upper extremities seem lengthened to attain the same purpose, and are more than naturally depressed towards the thighs.

In the pelvis itself, whether a new joint is formed or not, the old cotyloid cavity undergoes the same changes. It always assumes the form of an isosceles triangle, whose base is represented by the notch at its anterior and inferior border; nothing remains of either the cotyloid ligament or the cartilage which formerly lined the greater part of the cavity; all is rude, irregular, and filled up by fat. The cause of the assumption of this triangular form seems to be as follows: when the head of the femur is forced out of the acetabulum, the capsular ligament in the part which retains its attachments both to the ilium and to the femur, is rendered extremely tense; and this force, continually acting, tends to produce a change of form in the bone by pressing the superior and inferior walls of the acetabulum towards each other. At the same time also the psoas magnus and iliacus internus on the upper border of the cavity, and the obturator externus on its inferior and outer border, being put on the stretch and frequently acting, tend in the same way to produce a change in the form of the acetabulum; not indeed by the direct influence of their pressure, but by the change which it produces in the direction of the nutritive process.

After the displacement of the head of the femur, it moves freely between the glutei, in contact with the surface of the ilium; and hence the formation of a new acetabulum. The vessels of the periosteum of the ilium, or perhaps of the ilium itself after its periosteum is worn through, throw out plastic lymph, which forms the basis of the new cavity, and in which first cartilage and then bone is deposited. This however takes place only when the capsule has been so torn as to permit the head of the femur to pass out of it and come in contact with the surface of the ilium. When this has not occurred, and the capsule has intervened between the two bones, then, if any impression at all be made in the ilium, it is only by a yielding and bending inwards of its ala, or by a gradual absorption of its external layers; but never, as far as Dr. Vrolik (who has specimens of all these changes) has seen, by a production of new bone.

The whole of the os innominatum also undergoes a change of form. The action of the gluteus medius being almost entirely destroyed, and its antagonist, the iliacus internus, being stretched and enabled by its position to act with more than usual power, the ilium, deprived of the force exerted upon it by the contractions of the former, is constrained to follow the action of the latter; and yielding to this force, it bends inwards, and instead of having an oblique direction from without inwards, it becomes almost vertical. In like manner and at the same time, the action of the muscles on its exterior being lost, the lines and

*** This remark affords important evidence on the subject of the oblique position of the pelvis described by Naegele, whose work we reviewed in Vol. XI. p. 167; it casts additional doubt on all his cases in which the state of the spine was unknown.**

processes for their attachment are gradually effaced; its internal surface acquires a concave form, and the brim of the pelvis is rounded off so as to render the boundary between the two pelvic cavities indistinct. The crest of the ilium also loses almost all its curve and becomes nearly rectilinear; and the notch between the superior and inferior anterior spines is partially effaced and appears longer than that on the opposite side.

The whole of the os innominatum becomes thinner and lighter. The horizontal ramus of the os pubis is longer than that on the opposite side, and at its junction with the ilium it presents a deep groove where the iliacus and psoas have played over it with unusual force. The tuber ischii is turned outwards; and its ramus assumes the same direction, drawn thither by the altered action of the adductor magnus, the obturator internus, the gemelli, and the quadratus femoris, all of which, in the new position of the femur, pass much more obliquely upwards and outwards than they did before. Hence also the change in the foramen ovale, which becomes elongated and narrow; and in the sciatic notch, which is somewhat diminished in its size; and in the angle of the symphysis pubis, which becomes much less acute.

The effect of these changes is, as Camper showed, favorable to the size of the pelvic cavity. The partial obliteration of the brim makes the transverse and oblique diameters greater than usual; and the turning outwards of the tuberosity and ramus of the ischium increases the width of the inferior aperture; so that on the whole, though the antero-posterior diameter is somewhat lessened, the general dimensions of the pelvic cavity are enlarged by the whole series of these changes. This however is not generally the case in congenital luxations; in these, though the changes that take place are on the whole similar to those that follow accidental dislocations, yet the approximation of the sacrum and symphysis pubis, by the increased projection of the former, and the elevation of the latter by the powerful action of the recti abdominis muscles constantly pulling upon the unnaturally inclined pelvis, often tend so much to diminish the antero-posterior diameter as to render an accouchement difficult or impossible.

The rest of the work is occupied with two remarkable cases of this congenital luxation, some observations on the explanations that have been offered of the process, and a case in which the author believes that the dislocation of the femur resulted from an unnatural development of the mass of fat (the synovial gland) at the bottom of the acetabulum. To these however our space does not allow us to do more than refer as good examples of the diligence and accuracy with which the labours of anatomy are still carried on in Holland. It is to be lamented that their results are so little known and appreciated in this country; and we shall be glad if by these and some future notices we can assist in obtaining for the worthy successors of the best anatomists in Europe the European reputation which they merit.

